# Selective Inhibition of Membrane Type 1 Matrix Metalloproteinase Abrogates Progression of Experimental Inflammatory Arthritis: Synergy With Tumor Necrosis Factor Blockade

**DOI:** 10.1002/art.39414

**Published:** 2016-01-25

**Authors:** Kazuyo Kaneko, Richard O. Williams, Daniel T. Dransfield, Andrew E. Nixon, Ann Sandison, Yoshifumi Itoh

**Affiliations:** ^1^ Kennedy Institute of Rheumatology and University of OxfordOxfordUK; ^2^ Dyax CorporationBurlington Massachusetts; ^3^ Charing Cross Hospital and Imperial College LondonLondonUK

## Abstract

**Objective:**

In rheumatoid arthritis (RA), destruction of articular cartilage by the inflamed synovium is considered to be driven by increased activities of proteolytic enzymes, including matrix metalloproteinases (MMPs). The purpose of this study was to investigate the therapeutic potential of selective inhibition of membrane type 1 MMP (MT1‐MMP) and its combination with tumor necrosis factor (TNF) blockage in mice with collagen‐induced arthritis (CIA).

**Methods:**

CIA was induced in DBA/1 mice by immunization with bovine type II collagen. From the onset of clinical arthritis, mice were treated with MT1‐MMP selective inhibitory antibody DX‐2400 and/or TNFR‐Fc fusion protein. Disease progression was monitored daily, and serum, lymph nodes, and affected paws were collected at the end of the study for cytokine and histologic analyses. For in vitro analysis, bone marrow–derived macrophages were stimulated with lipopolysaccharide for 24 hours in the presence of DX‐2400 and/or TNFR‐Fc to analyze cytokine production and phenotype.

**Results:**

DX‐2400 treatment significantly reduced cartilage degradation and disease progression in mice with CIA. Importantly, when combined with TNF blockade, DX‐2400 acted synergistically, inducing long‐term benefit. DX‐2400 also inhibited the up‐regulation of interleukin‐12 (IL‐12)/IL‐23 p40 via polarization toward an M2 phenotype in bone marrow–derived macrophages. Increased production of IL‐17 induced by anti‐TNF, which correlated with an incomplete response to anti‐TNF, was abrogated by combined treatment with DX‐2400 in CIA.

**Conclusion:**

Targeting MT1‐MMP provides a potential strategy for joint protection, and its combination with TNF blockade may be particularly beneficial in RA patients with an inadequate response to anti‐TNF therapy.

Rheumatoid arthritis (RA) is a systemic inflammatory disease characterized by progressive infiltration of the joints by leukocytes, production of mediators of inflammation, and the eventual destruction of joints, including the cartilage and bone 1. The introduction of tumor necrosis factor (TNF) inhibitors has greatly improved the management of RA. However, there remains a need to develop more effective and longer‐lasting treatments for RA because a proportion of patients fail to respond to TNF inhibitors or their responsiveness is lost over time 2, 3. Approaches combining a TNF inhibitor and other approved biologic agents that target different immunomodulatory pathways, such as CTLA‐4 and interleukin‐1 (IL‐1), have shown no added efficacy but an increased risk of serious infections has been reported 4, 5, suggesting that it is important to identify a new combination partner that improves response to anti‐TNF therapy without increasing the risk of side effects.

During the progression of RA, the synovium becomes hyperplastic and locally invasive (commonly known as pannus), penetrating the surface of the cartilage and degrading its extracellular matrix 6. The cartilage extracellular matrix is primarily composed of fibrillar type II collagen and proteoglycan aggrecan, the degradation of which by pannus is associated with increased activity of proteolytic enzymes, including matrix metalloproteinases (MMPs) and aggrecanases 7. Early aggrecanase‐mediated loss of aggrecan from cartilage can be reversed, but after the induction of MMP‐mediated breakdown of collagen, cartilage damage becomes irreversible and leads to joint dysfunction 8. Thus, collagen degradation by MMPs is thought to be a critical step in the progression of joint damage.

The RA synovium consists of 2 major resident cell types, macrophage‐like synoviocytes and fibroblast‐like synoviocytes (FLS), along with recruited inflammatory cells, such as T cells, macrophages, B cells, dendritic cells, and mast cells 9. Among these cells, FLS and macrophages are the major sources of MMPs. FLS activated through cellular interactions and soluble factors produce MMP‐1, MMP‐2, MMP‐13, and membrane type 1 MMP (MT1‐MMP; also known as MMP‐14), which can degrade type II collagen. Macrophages also produce MMP‐1, MMP‐2, and MT1‐MMP 7, 10. However, the precise functions of these MMPs in cartilage degradation remain elusive. The failure of broad‐spectrum MMP inhibitors in clinical trials of cancer and RA 11 emphasizes the importance of targeting specific enzymes.

Among these collagenolytic MMPs, MT1‐MMP is a type I transmembrane proteinase that is expressed on the cell surface and the only collagenase that directly promotes cellular invasion into 3‐dimensional collagen matrices 12. Our previous work showed that MT1‐MMP is highly expressed in FLS and macrophages at the cartilage–pannus junction in the joints of patients with RA and promotes the invasion of RA FLS into cartilage in vitro 13. Similar results were obtained by Sabeh et al 14, who demonstrated that silencing MT1‐MMP, but not MMP‐1, MMP‐2, or MMP‐13, inhibited cartilage invasion by RA synoviocytes 14. The findings of these studies suggest that MT1‐MMP is a key enzyme in cartilage invasion by pannus in RA.

We used the collagen‐induced arthritis (CIA) mouse model in the present study to determine whether MT1‐MMP is a potential therapeutic target for joint damage in RA. We demonstrated that selective inhibition of MT1‐MMP protects joints from cartilage damage and disease progression and enhances the response to anti‐TNF treatment in established CIA.

## PATIENTS AND METHODS

### Collagen‐induced arthritis

CIA was induced in male DBA/1 mice (11–13 weeks old) by immunization with bovine type II collagen, as previously described 15. From the onset of clinical arthritis, mice were treated intraperitoneally with either IgG isotype control, anti–MT1‐MMP inhibitory antibody DX‐2400 (20 or 40 mg/kg), TNFR‐Fc fusion protein (2 or 8 mg/kg), or a combination of DX‐2400 (20 mg/kg) and TNFR‐Fc (2 mg/kg) every other day for 10 days. DX‐2400 and IgG isotype control are recombinant human IgG1 expressed in Chinese hamster ovary cells as described previously 16. TNFR‐Fc (etanercept) was obtained from the hospital pharmacy.

To avoid cage effects, treatment groups were randomized between cages. As individual mice developed clinical arthritis, they were assigned to different treatment groups in a sequential manner, irrespective of their cage. For long‐term withdrawal studies, mice were treated for the first 5 days, and disease progression was monitored for 20 days after arthritis onset. Mice were monitored daily for arthritis. Paw thickness was measured with calipers. Visual assessment of arthritis severity was performed in a nonblinded manner and scored on a scale of 0–3, where 0 = normal, 1 = slight swelling and/or erythema, 2 = pronounced swelling, and 3 = ankylosis. All 4 limbs were scored, and the results were summed, giving a maximum score of 12. Serum and paw samples were collected at the end of the study. We confirmed that injection of control IgG had a negligible effect on disease severity as compared with that in untreated mice.

All experimental procedures were approved by the local Ethics Review Process Committee and the UK Home Office.

### Histologic assessment of joints

At the end of study, the first affected paws were harvested, fixed in 10% neutral buffered formalin, and then decalcified in 10% EDTA for 3 weeks before standard processing for paraffin embedding. Sections (5 μm) were cut and stained with Safranin O for microscopic evaluation of the tarsometatarsal joints, which was performed in a blinded manner. Cartilage thickness was measured at 140‐μm intervals using ImageJ software (National Institutes of Health) and calculated by averaging at least 10 measurements per image. Bone erosion was defined as demarcated defects in cartilage or bone associated with pannus invasion and was scored visually on a scale of 0–4, where 0 = none, 1 = minimal (1–2 sites of surface erosion), 2 = mild (at least 3 sites of surface erosion), 3 = moderate (discrete foci of erosion), and 4 = marked (large erosions extending into the marrow space).

For immunostaining of MT1‐MMP, sections were deparaffinized, rehydrated, and subjected to antigen retrieval in 0.1*M* citrate buffer (pH 6.0) overnight at 60°C. After blocking for 1 hour with 10% goat serum and 1% bovine serum albumin, the sections were incubated for 2 hours at room temperature with rabbit anti–MT1‐MMP monoclonal antibody (1:250 dilution; Epitomics), followed by incubation for 1 hour at room temperature with biotinylated secondary antibody (1:300 dilution). Staining was visualized with the use of Vectastain ABC kits and diaminobenzidine substrate (Vector) according to the manufacturer's instructions. Slides were subsequently counterstained with hematoxylin.

### Cell culture

Human FLS were isolated from synovial tissues obtained from RA patients, as previously described 13. Inguinal lymph nodes from mice were excised 10 days after the onset of CIA. Cells were dissociated and cultured at a density of 2 × 10^6^ cells/ml in RPMI 1640 supplemented with 10% fetal bovine serum (FBS), 50 units/ml of penicillin/streptomycin, and 50 μ*M* 2‐mercaptoethanol. Cells were left unstimulated or were stimulated with 100 ng/ml of anti‐CD3 monoclonal antibody (145‐2C11; eBioscience), and supernatants were collected for cytokine analysis at 48 hours after stimulation.

For generation of bone marrow–derived macrophages (BMMs), bone marrow cells were flushed from the tibias and femurs of 7–12‐week‐old male DBA/1 mice. Erythrocytes were depleted using an Erythrocyte Lysing kit (R&D Systems), and the remaining cells were cultured in RPMI 1640 supplemented with 10% FBS, 50 units/ml of penicillin/streptomycin, and 50 μ*M* 2‐mercaptoethanol containing 50 ng/ml of macrophage colony‐stimulating factor (PeproTech). The medium was changed on day 3. BMMs were harvested on day 6 using a nonenzymatic cell dissociation solution (Sigma‐Aldrich) and were plated in a 96‐well plate (5 × 10^4^ cells/well) or a 48‐well plate (3 × 10^5^ cells/well). The next day, cells were treated with 10 n*M* DX‐2400 and/or TNFR‐Fc, with or without lipopolysaccharide (LPS; 1 ng/ml), and at 24 hours after treatment, supernatants and total RNA, which was extracted using an RNeasy kit (Qiagen), were collected.

### Isolation of CD4+ T cells and B cells

Single‐cell suspensions were prepared from the spleens of 7–12‐week‐old DBA/1 mice. After erythrocyte depletion, CD4+ T cells and B cells were purified by positive selection using magnetic beads (Dynabeads; Invitrogen) according to the manufacturer's instructions. Isolated cells were subjected to RNA extraction using an RNeasy kit.

### Collagen film degradation assay

Type I collagen thin films were prepared as previously described 13. FLS (1 × 10^5^ cells/well) were cultured for 4 days on collagen film in Dulbecco's modified Eagle's medium (DMEM) supplemented with 10% FBS in the presence or absence of 100 or 500 n*M* DX‐2400 or 10 μ*M* GM6001 (Elastin Products). Cells were removed by treatment with trypsin–EDTA (Sigma‐Aldrich), and the films were fixed with 3% paraformaldehyde and stained with Coomassie blue. Images were captured using a 20× objective lens.

### Cartilage invasion assay

Cartilage invasion assay was carried out as previously described 13. FLS were cultured for 4 weeks on bovine nasal cartilage explants in DMEM supplemented with 2% FBS in the presence or absence of 100 or 500 n*M* DX‐2400 or 10 μ*M* GM6001. Cartilage explants were then fixed in 4% formalin and embedded in paraffin. Sections were cut at 5 μm and stained with mouse anti–MT1‐MMP monoclonal antibody (222‐1D8; Daiichi Fine Chemical Company), followed by counterstaining with hematoxylin.

### Measurement of cytokine and cartilage oligomeric matrix protein (COMP)

IL‐17, interferon‐γ (IFNγ), and IL‐10 were measured using DuoSet enzyme‐linked immunosorbent assay (ELISA) kits (R&D Systems). For measurement of IL‐12/IL‐23 p40, we used an IL‐12/IL‐23 total p40 ELISA kit (eBioscience). Serum COMP levels were determined using an animal COMP ELISA kit (AnaMar).

### Real‐time reverse transcription–polymerase chain reaction (RT‐PCR)

Total RNA was reverse transcribed into complementary DNA (cDNA) using a high‐capacity cDNA reverse transcription kit (Invitrogen) according to the manufacturer's instructions. Messenger RNA (mRNA) expression levels were analyzed by real‐time RT‐PCR using the following TaqMan probes: Mm00485054_m1 for MT1‐MMP, Mm00440502_m1 for type 2 nitric oxide synthase (NOS2), and Mm00475988_m1 for arginase 1. GAPDH served as an endogenous control.

### Statistical analysis

Comparisons between 2 groups were performed using an unpaired 2‐tailed *t*‐test. For experiments involving multiple groups, one‐way analysis of variance (ANOVA) followed by Turkey's multiple comparison test was used, except for analysis of clinical scores and paw thicknesses, which used two‐way ANOVA followed by Dunnett's multiple comparison test. Calculations were made using GraphPad Prism 6 software. *P* values less than 0.05 were considered significant.

## RESULTS

### Selective inhibition of MT1‐MMP and amelioration of CIA

**Figure 1:**
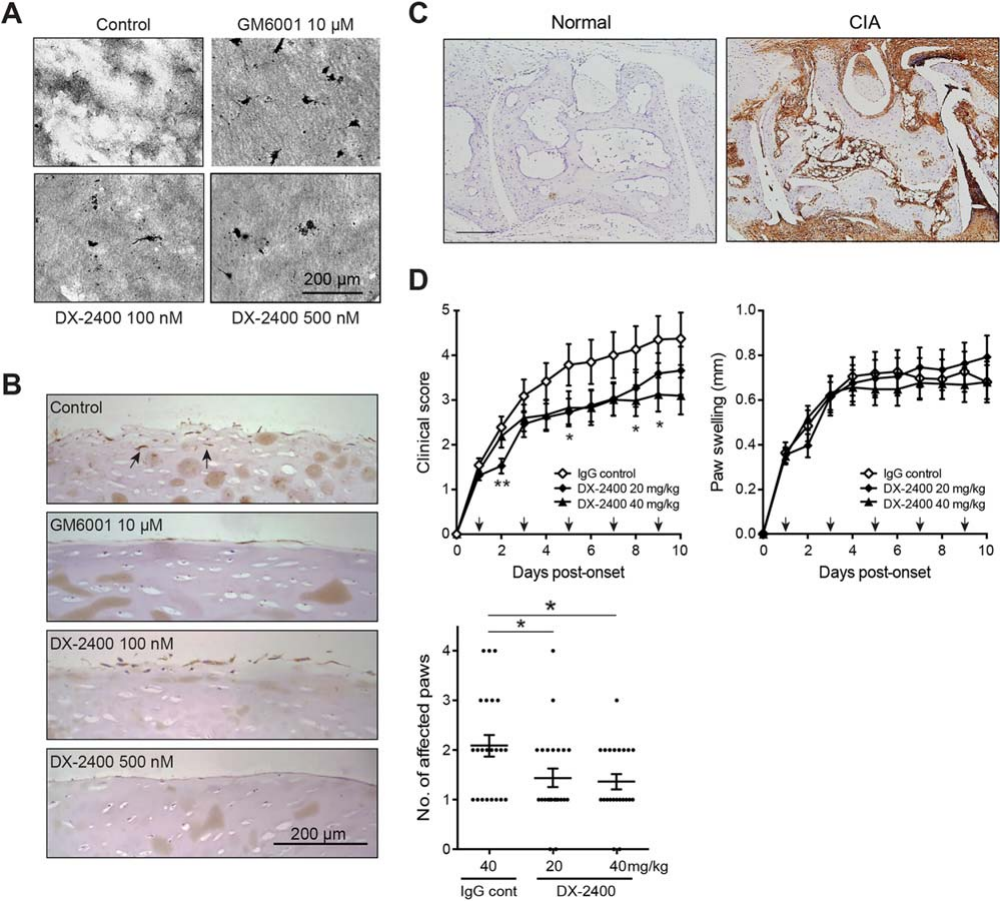
Reduced progression of arthritis following selective inhibition of membrane type 1 matrix metalloproteinase (MT1‐MMP) in mice with collagen‐induced arthritis (CIA). **A,** Collagen film degradation by human rheumatoid arthritis (RA) fibroblast‐like synoviocytes (FLS) in the absence or presence of 100 n*M* or 500 n*M* DX‐2400, 10 μ*M* GM6001, or buffer (control). Clear, unstained areas indicate collagen degradation. **B,** Invasion of human RA FLS into the cartilage in the presence of buffer alone (control), 10 μ*M* GM6001, or 100 n*M* or 500 n*M* DX‐2400. **Arrows** show invading FLS. **C,** Immunostaining for MT1‐MMP in the tarsometatarsal joints of a normal mouse and a mouse with CIA (10 days after arthritis onset). Bar = 200 μm. **D,** Clinical scores, paw swelling (first affected paw), and number of affected paws 10 days after the onset of arthritis in mice with CIA treated with IgG control or with 20 or 40 mg/kg of DX‐2400. **Arrows** indicate injection times. For the clinical scores and paw swelling data, values are the mean ± SEM of 22–23 mice per group. For the number of affected paws, each symbol represents an individual mouse; horizontal lines with bars show the mean ± SEM of 22–23 mice per group. **∗** = *P* < 0.05 for the indicated comparison or versus IgG control; **∗∗** = *P* < 0.01 versus IgG control. Color figure can be viewed in the online issue, which is available at http://onlinelibrary.wiley.com/journal/doi/10.1002/art.39414/abstract

Selective inhibition of MT1‐MMP by an MT1‐MMP inhibitory antibody, DX‐2400, markedly inhibited the degradation of collagen film and the cartilage invasion by human RA FLS in a dose‐dependent manner (Figures 1A and B). To assess the effect of DX‐2400 on cartilage degradation in vivo, we examined mice with CIA, a widely used murine model of RA. MT1‐MMP was highly expressed in the joints of mice with CIA, while negligible expression was observed in normal joints (Figure 1C). In general, CIA initially affected 1 paw and then progressed to affect other paws during the course of the disease. Treatment with DX‐2400 moderately reduced the clinical score, principally by reducing the spread of the disease to unaffected joints, rather than by ameliorating the already affected joints (Figure 1D).

**Figure 2:**
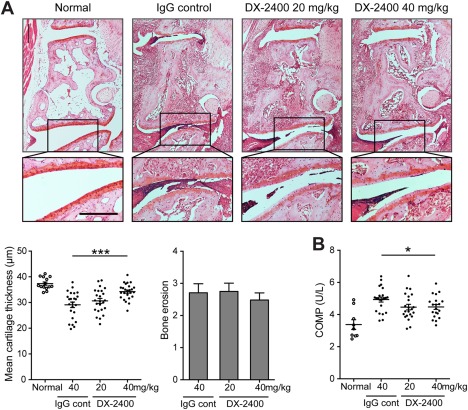
Prevention of cartilage degradation following selective inhibition of membrane type 1 matrix metalloproteinase in mice with collagen‐induced arthritis (CIA). **A,** Histologic analysis of a joint from a normal mouse and arthritic joints from mice with CIA treated for 10 days with IgG control or with 20 or 40 mg/kg of DX‐2400. Images at the top are representative images of Safranin O–stained sections of the tarsometatarsal joints. Images at the bottom are higher‐magnification views of the boxed areas in the images at the top. Bar = 200 μm. The mean cartilage thickness (bottom left) and bone erosion scores (bottom right) in the tarsometatarsal joints of mice from the same treatment groups are also shown (n = 22–23 mice per group). For the mean cartilage thickness, each symbol represents an individual mouse; horizontal lines with bars show the mean ± SEM. For the bone erosion scores, values are the mean ± SEM. **∗∗∗** = *P* < 0.001. **B,** Serum levels of cartilage oligomeric matrix protein (COMP) in normal mice and mice with CIA treated for 10 days with IgG control or with 20 or 40 mg/kg of DX‐2400 (n = 22–23 mice per group). Each symbol represents an individual mouse; horizontal lines with bars show the mean ± SEM. **∗** = *P* < 0.05.

Despite the lack of effect of DX‐2400 on swelling (Figure 1D), histologic analysis of the joints from the first affected paws (Figure 2A) showed that the higher dose of DX‐2400 led to markedly better preservation of cartilage structure and significantly less degradation of cartilage as compared with the IgG control. This observation was supported by the fact that serum levels of COMP, a marker of cartilage destruction 17, were also significantly lower in this group as compared to the IgG control group (Figure 2B). However, DX‐2400 treatment had minimal effect on bone erosion (Figure 2A).

Thus, selective inhibition of MT1‐MMP effectively prevented cartilage degradation and arthritis progression but had minimal effects on swelling and bone erosion in joints with established arthritis.

### Synergistic effects of combined inhibition of MT1‐MMP and TNF

**Figure 3:**
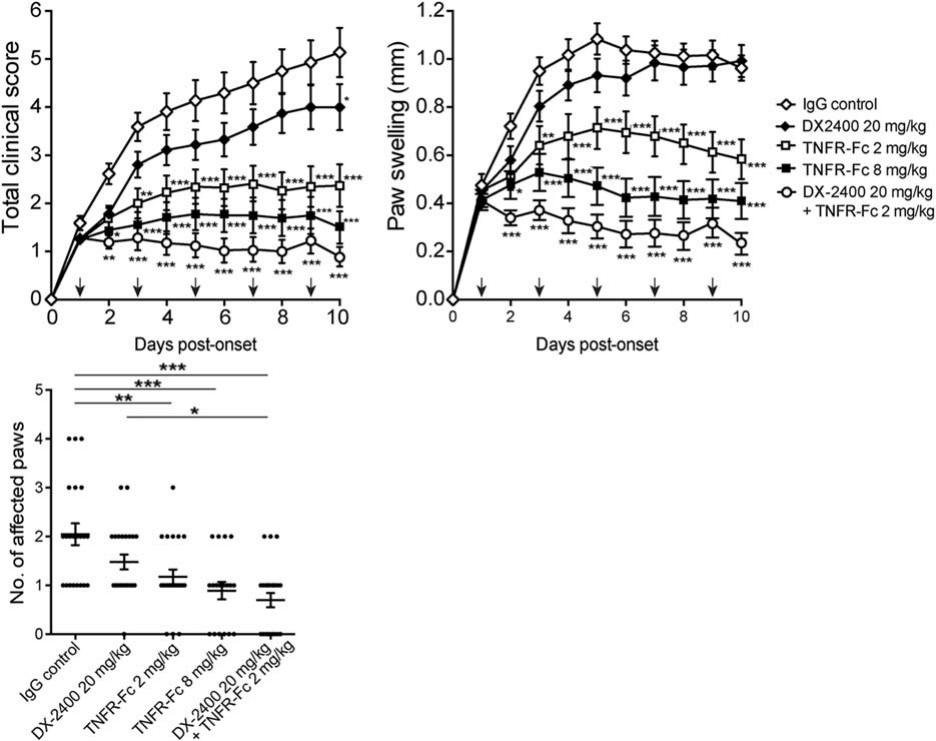
Synergistic enhancement of the efficacy of anti–tumor necrosis factor (anti‐TNF) treatment following selective inhibition of membrane type 1 matrix metalloproteinase in mice with collagen‐induced arthritis (CIA). Mice with CIA were treated with IgG control, 20 mg/kg of DX‐2400, 2 or 8 mg/kg of TNFR‐Fc fusion protein, or 20 mg/kg of DX‐2400 plus 2 mg/kg TNFR‐Fc for 10 days from the onset of arthritis (n = 18–23 mice per group). Clinical scores (left) and paw swelling (first affected paw) (right) are shown at the top. **Arrows** indicate injection times. Values are the mean ± SEM. The number of affected paws 10 days after the onset of arthritis is shown at the bottom. Each symbol represents an individual mouse; horizontal lines with bars show the mean ± SEM. **∗** = *P* < 0.05; **∗∗** = *P* < 0.01; **∗∗∗** = *P* < 0.001 for the indicated comparisons or versus IgG control.

Since TNF plays an important role in joint inflammation 18 as well as osteoclast differentiation and activation 19, 20, we next assessed whether the combination of DX‐2400 and a TNF inhibitor (soluble TNFR‐Fc) might provide an additive or synergistic effect in CIA. As shown in Figure 3, suboptimal dosing with TNFR‐Fc (2 mg/kg) moderately but significantly reduced both the clinical score and paw swelling, which were further reduced by optimal dosing with TNFR‐Fc (8 mg/kg). Suboptimal and optimal dosing has been established in previous studies 21, 22. The combination of DX‐2400 and TNFR‐Fc profoundly reduced both the clinical score and paw swelling in a synergistic manner, and the effect was greater than optimal dosing with TNFR‐Fc alone. Although arthritis progression was inhibited in all groups, the arthritis was completely resolved in 43% of mice given the combined treatment, as compared to 4% and 13% of mice given DX‐2400 and TNFR‐Fc alone, respectively.

**Figure 4:**
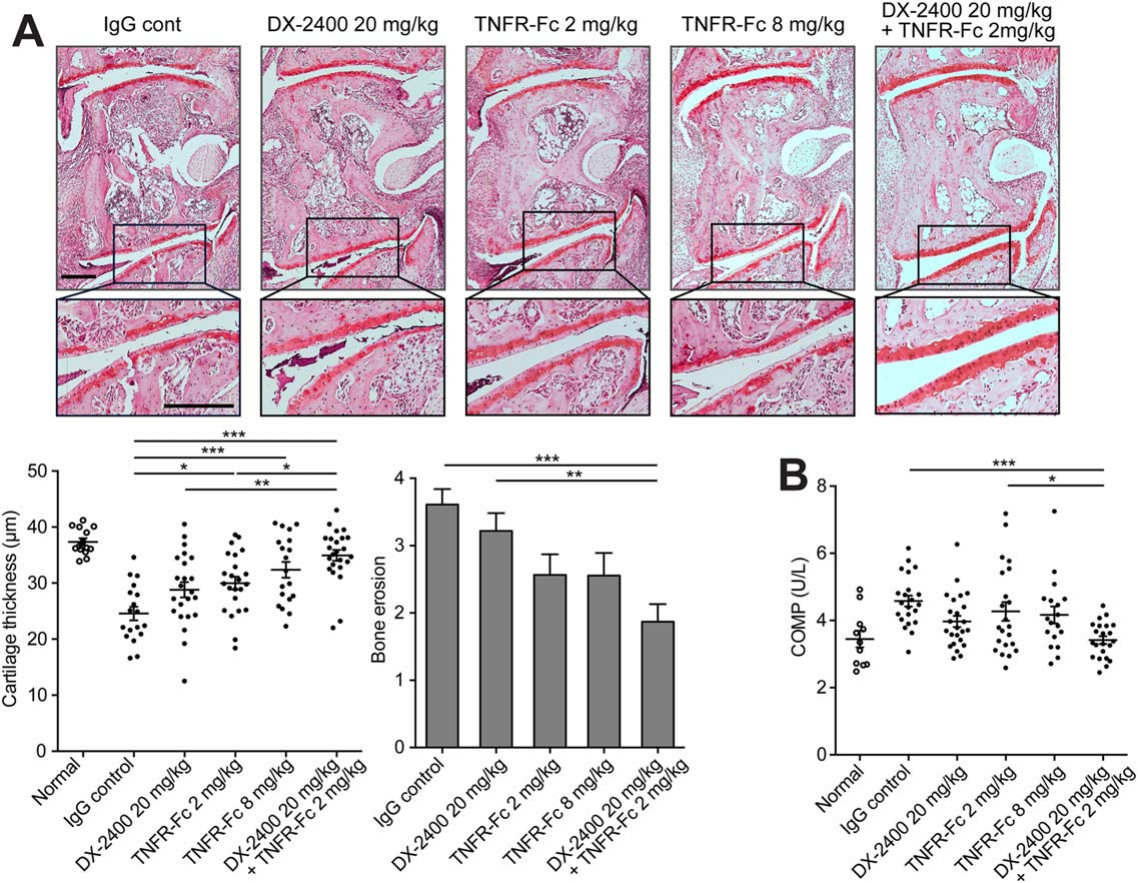
Prevention of both cartilage degradation and bone erosion following combined inhibition of membrane type 1 matrix metalloproteinase and tumor necrosis factor (TNF) in mice with collagen‐induced arthritis (CIA). **A,** Histologic analysis of arthritic joints from mice with CIA treated with IgG control, 20 mg/kg of DX‐2400, 2 or 8 mg/kg of TNFR‐Fc fusion protein, or 20 mg/kg of DX‐2400 plus 2 mg/kg of TNFR‐Fc for 10 days from the onset of arthritis. Images at the top are representative images of Safranin O–stained sections of the tarsometatarsal joints. Images at the bottom are higher‐magnification views of the boxed areas in the images at the top. Bar = 200 μm. The mean cartilage thickness (bottom left) and bone erosion scores (bottom right) in the tarsometatarsal joints of mice from the same treatment groups are also shown (n = 18–23 mice per group). For the mean cartilage thickness, each symbol represents an individual mouse; horizontal lines with bars show the mean ± SEM. For the bone erosion scores, values are the mean ± SEM. **B,** Serum levels of cartilage oligomeric matrix protein (COMP) in normal mice and mice with CIA in the same treatment groups (n = 22–23 mice per group). Each symbol represents an individual mouse; horizontal lines with bars show the mean ± SEM. **∗** = *P* < 0.05; **∗∗** = *P* < 0.01; **∗∗∗** = *P* < 0.001. Color figure can be viewed in the online issue, which is available at http://onlinelibrary.wiley.com/journal/doi/10.1002/art.39414/abstract

In terms of joint damage, cartilage degradation was significantly inhibited by treatment with TNFR‐Fc alone in a dose‐dependent manner, and this effect was synergistically enhanced when TNFR‐Fc was combined with DX‐2400 (Figure 4A). Serum COMP levels and bone erosion were also significantly reduced in the combined treatment group, but not in the groups receiving DX‐2400 or TNFR‐Fc alone (Figures 4A and B).

**Figure 5:**
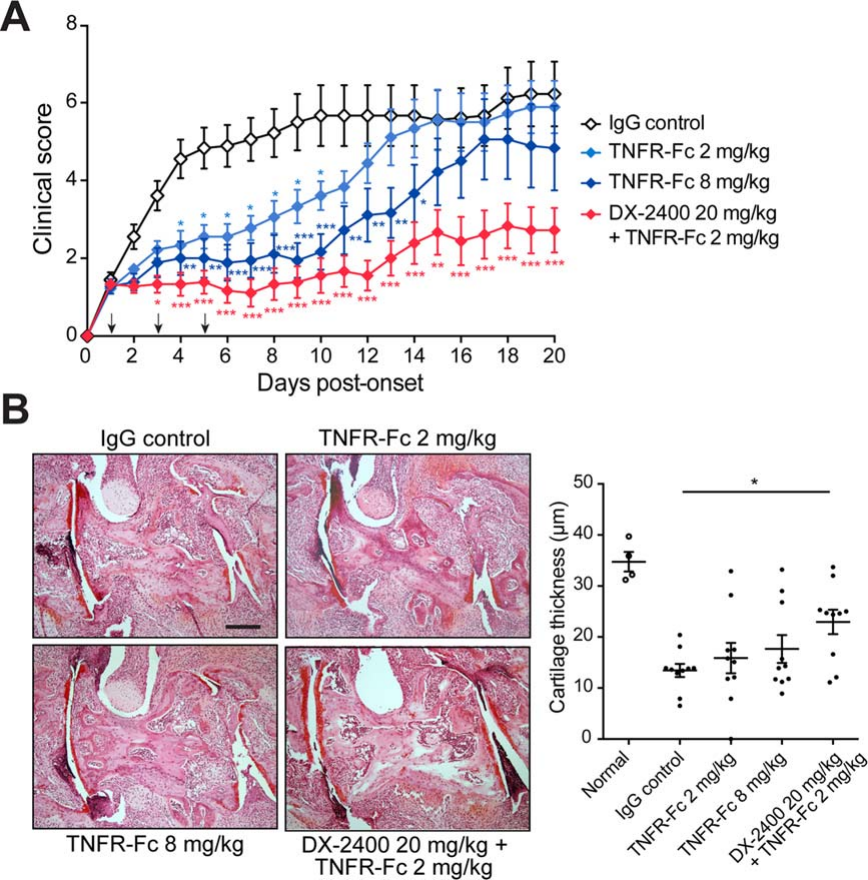
Induction of long‐term benefit following combined inhibition of membrane type 1 matrix metalloproteinase and tumor necrosis factor (TNF) in mice with collagen‐induced arthritis (CIA). Mice with CIA were treated with IgG control, 2 or 8 mg/kg of TNFR‐Fc fusion protein, or 20 mg/kg of DX‐2400 plus 2 mg/kg of TNFR‐Fc for the first 5 days after the onset of arthritis (n = 10 mice per group). **A,** Clinical scores in the 4 treatment groups. **Arrows** indicate injection times. Values are the mean ± SEM. **∗** = *P* < 0.05; **∗∗** = *P* < 0.01; **∗∗∗** = *P* < 0.001 versus IgG control. **B,** Histologic analysis of arthritic joints 20 days after disease onset. Images at the left are representative images of Safranin O–stained sections of the tarsometatarsal joints. Bar = 200 μm. The mean cartilage thickness in the tarsometatarsal joints of normal mice and mice in the same 4 treatment groups is shown at the right. Each symbol represents an individual mouse; horizontal lines with bars show the mean ± SEM. **∗** = *P* < 0.05. Color figure can be viewed in the online issue, which is available at http://onlinelibrary.wiley.com/journal/doi/10.1002/art.39414/abstract

We also examined the effect on long‐term disease progression of withdrawal of DX‐2400 and TNFR‐Fc. The results showed that in the combined treatment group, arthritis severity remained stable after stopping treatment and remained significantly lower than that in the IgG control group at least until 15 days after cessation of treatment, whereas with TNFR‐Fc treatment alone, the disease relapsed shortly after stopping treatment and reached the control level (Figure 5A). Cartilage degradation was also significantly inhibited at 15 days after cessation of treatment in mice given combined treatment (Figure 5B).

### Influence of MT1‐MMP on macrophage function

**Figure 6:**
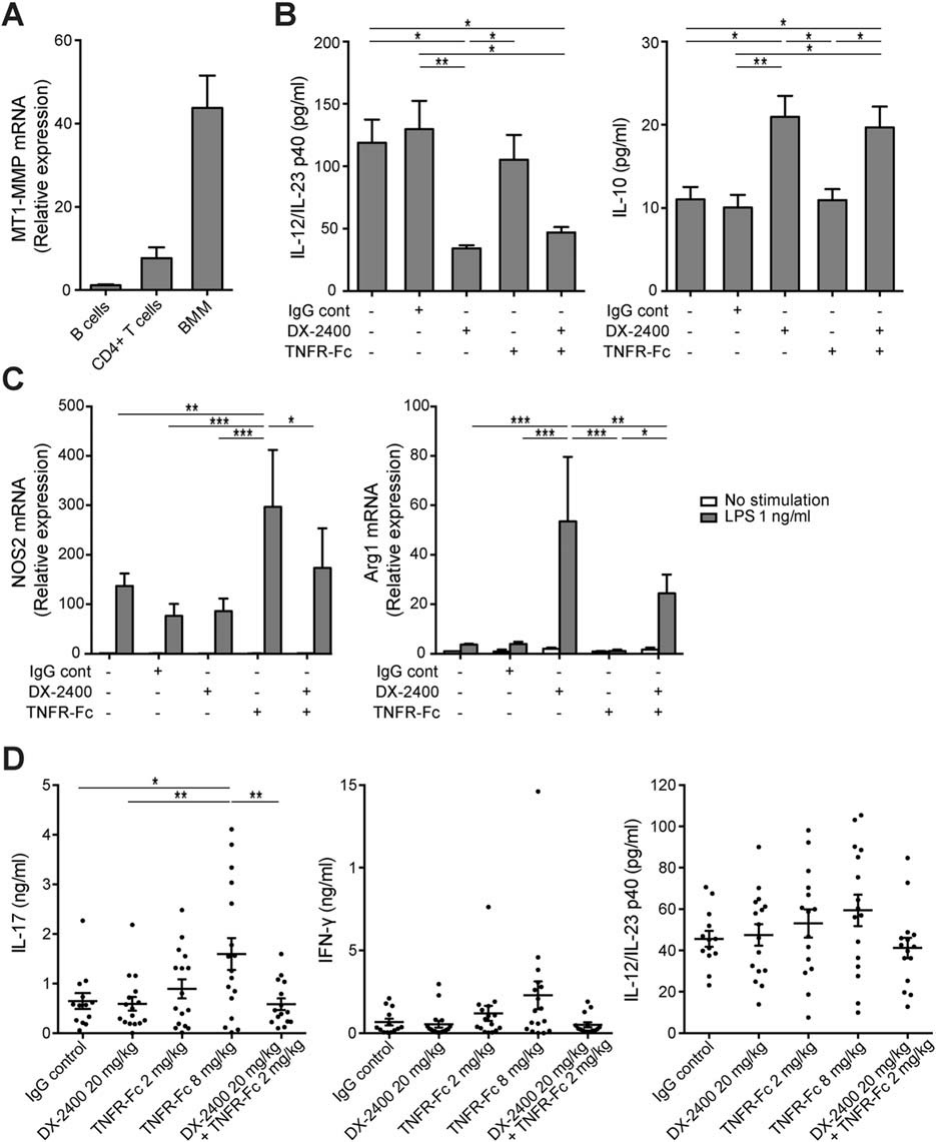
Promotion of M2 macrophage polarization and inhibition of the Th1/Th17 cell response exerted by treatment with anti–tumor necrosis factor (anti‐TNF) following selective inhibition of membrane type 1 matrix metalloproteinase (MT1‐MMP) in mice with collagen‐induced arthritis (CIA). **A,** Expression of mRNA for MT1‐MMP in B cells, CD4+ T cells, and bone marrow–derived macrophages (BMMs) from mice with CIA. Expression levels were calculated relative to those in B cells. **B,** Release of interleukin‐12 (IL‐12)/IL‐23 p40 (left) and IL‐10 (right) from BMMs following 24 hours of stimulation with IgG control or with 1 ng/ml of lipopolysaccharide (LPS) in the presence or absence of 10 n*M* DX‐2400 and/or TNFR‐Fc. **C,** Expression of mRNA for M1 (nitric oxide synthase 2 [NOS2]) (left) and M2 (arginase 1 [Arg1]) (right) macrophage markers in BMMs following 24 hours of stimulation with IgG control or with 10 n*M* DX‐2400 and/or TNFR‐Fc with or without 1 ng/ml of LPS. Expression levels were calculated relative to those in untreated cells. Values in **A–C** are the mean ± SEM of 3–4 mice per group. **D,** Release of IL‐17, interferon‐γ (IFNγ), and IL‐12/IL‐23 p40 from lymph node cells stimulated for 48 hours with anti‐CD3 monoclonal antibody. Lymph node cells were obtained from mice with CIA treated with IgG control, 20 mg/kg of DX‐2400, 2 or 8 mg/kg of TNFR‐Fc fusion protein, or 20 mg/kg of DX‐2400 plus 2 mg/kg of TNFR‐Fc 10 days after the onset of arthritis (n = 13–17 mice per group). Each symbol represents an individual mouse; horizontal lines with bars show the mean ± SEM. **∗** = *P* < 0.05; **∗∗** = *P* < 0.01; **∗∗∗** = *P* < 0.001.

Synergy between inhibition of MT1‐MMP and TNF is of great interest because it suggests that an MT1‐MMP inhibitor would be effective in anti‐TNF nonresponders. Previous work in mice has shown that despite reduced arthritis severity, inhibition of TNF leads to an expansion of peripheral Th1/Th17 cells via up‐regulation of the common p40 subunit of IL‐12/IL‐23 in myeloid cells 23. Subsequent studies have shown that an increase in Th17/IL‐17 and p40 levels correlates with an incomplete response to anti‐TNF treatment in patients with RA 24, 25. Since macrophages also express high levels of MT1‐MMP (Figure 6A) and are the primary source of TNF 26, 27, we examined whether DX‐2400 could modulate cytokine production by macrophages in vitro. When BMMs were stimulated for 24 hours with LPS in the presence of DX‐2400 and/or TNFR‐Fc, DX‐2400 was found to significantly reduce p40 production but increase IL‐10 production regardless of the presence or absence of TNFR‐Fc (Figure 6B). Without LPS stimulation, neither p40 nor IL‐10 was detected (data not shown). The failure to obtain increased p40 production with TNFR‐Fc treatment was possibly because its production reached a maximum level under LPS stimulation alone.

The cytokine production profile in macrophages depends on their phenotype; classically activated M1 macrophages express IL‐12^high^/IL‐10^low^, whereas alternatively activated M2 macrophages express IL‐12^low^/IL‐10^high^ 28. Analysis of mRNA expression of M1 (NOS2) and M2 (arginase 1) markers in BMMs treated with LPS showed that DX‐2400 significantly up‐regulated arginase 1 expression, whereas TNFR‐Fc significantly up‐regulated NOS2 expression. With the combination of DX‐2400 and TNFR‐Fc, the TNFR‐Fc–induced up‐regulation of NOS2 expression was counteracted, but the arginase 1 expression remained significantly higher, albeit to a lesser extent than that with DX‐2400 alone (Figure 6C). This suggests that DX‐2400 could divert the polarization of macrophages toward the antiinflammatory M2 phenotype, resulting in a reduction of p40 production.

### Selective inhibition of MT1‐MMP and abrogation of the anti‐TNF–induced up‐regulation of IL‐17 and IFNγ

To determine whether the M1‐to‐M2 shift induced by DX‐2400 in vitro had an additive effect on T cell activity in vivo, we next isolated lymph node cells from mice with CIA 10 days after the start of treatment and stimulated them with anti‐CD3 to determine cytokine production. Consistent with previous reports 23, 24, 29, cells isolated from mice treated with TNFR‐Fc alone produced more IL‐17 and IFNγ and in a dose‐dependent manner than did those treated with IgG control. Treatment with DX‐2400 alone did not alter the production of either IL‐17 or IFNγ, but interestingly, the TNFR‐Fc–induced increase in IL‐17 and IFNγ was not seen when combined with DX‐2400 (Figure 6D). A similar trend was observed after stimulation with type II collagen (data not shown). Additionally, there was a trend toward increased production of p40 in cells isolated from mice treated with TNFR‐Fc alone, but not those treated with DX‐2400 and TNFR‐Fc (Figure 6D).

## DISCUSSION

The present study demonstrated 2 important proteolytic roles of MT1‐MMP in RA: destruction of cartilage in joints with established RA and migration of pathogenic cells to unaffected joints. The inhibition of joint damage is a major goal of RA therapy. However, some patients who achieved clinical remission still experience progression of joint damage 30. In addition, current therapy, including TNF inhibition, does not abrogate cartilage damage as much as it does bone erosion 31, 32, 33. Hence, targeting MT1‐MMP could represent a therapeutically useful strategy for minimizing cartilage damage. Consistent with earlier studies indicating that FLS are primarily responsible for cartilage damage in RA 9, we found that expression of MT1‐MMP was remarkably high in synovium and that selective inhibition of MT1‐MMP prevented the invasion of FLS into the cartilage (Figure 1). This suggests that reducing synovial invasiveness is likely to be a mechanism by which MT1‐MMP blockade inhibits cartilage damage. MT1‐MMP expression was also detected in chondrocytes, but cartilage degradation around MT1‐MMP–positive chondrocytes was not observed (Figure 1). Thus, at least in the experimental setting of the present study, the impact of MT1‐MMP blockade in chondrocytes is likely to be minimal.

As well as joint damage, continuous spreading of disease is a key feature of RA, although the underlying mechanisms remain largely unknown. Infiltration of circulating inflammatory cells into unaffected joints has long been speculated to be a potential cause. Indeed, a recent study suggested that activated FLS may be able to migrate from an inflamed joint to distant unaffected joints, leading to cartilage degradation 34. Further studies are required to identify the cell types involved in MT1‐MMP–mediated spreading of arthritis, but evidence suggests that migration of FLS and monocytes depends on MT1‐MMP 13, 35. It is therefore possible that these cells use MT1‐MMP to migrate into unaffected joints.

In contrast to the clear protective effect on cartilage degradation, selective inhibition of MT1‐MMP alone had minimal effects on bone erosion in established arthritis, despite MT1‐MMP being highly expressed in osteoclasts 36. While it has been suggested that MT1‐MMP functions in the migration and attachment of osteoclasts 37, cysteine protease cathepsin K appears to be the critical proteinase for osteoclastic collagen degradation 38, which may explain why inhibition of MT1‐MMP is not sufficient to inhibit bone erosion. Our analysis is based on an observational scoring system, however, and a more detailed quantitative analysis of bone erosion, such as micro–computed tomographic imaging, will be needed to draw conclusions about the effect of selective inhibition of MT1‐MMP in bone erosion.

The results of the present study revealed that combined inhibition of MT1‐MMP and TNF synergistically inhibits not only joint damage, but also synovial inflammation. These effects were maintained after discontinuation of treatment (Figure 5). If applied to humans, this combined therapeutic approach could allow for a reduction in both the dosage and frequency of administration of TNF inhibitors and possibly the induction of drug‐free remission, particularly in the early stages of RA. While further studies are required to elucidate the mechanisms involved, this synergistic effect is unlikely to be due to decreased levels of MT1‐MMP, since neither TNF nor IL‐1 directly affects the expression of MT1‐MMP in FLS (Kaneko K, et al: unpublished observations). However, anti‐TNF therapy can reduce the production of other MMPs, including MMP‐1 and MMP‐3 7, 39, which may contribute to the synergistic effect. It has also been reported that anti‐TNF therapy inhibits the recruitment of lymphocytes and monocytes into inflamed joints 40. The additional inhibition of MT‐1‐MMP may lead to the blockage of a whole range of pathogenic cells from migrating into the joint, thus preventing the progression of arthritis.

In addition to the effect on cell migration and invasion, we unexpectedly found that anti–MT1‐MMP antibody modifies cytokine production by macrophages toward an antiinflammatory profile, likely by driving polarization toward an M2 phenotype. How the blocking of MT1‐MMP alters macrophage polarization remains to be elucidated, but the mechanism may be mediated through the noncatalytic properties of MT1‐MMP, since neither the broad‐spectrum small‐molecule MMP inhibitor GM6001 nor tissue inhibitor of metalloproteinases 2 showed an effect similar to that of DX‐2400 on macrophage polarization (data not shown). This is supported by recent evidence suggesting that MT1‐MMP modulates cellular functions in a catalytic activity–dependent and –independent manner, such as through molecular interactions 41. The observed effects on macrophages were further extended in the CIA model, revealing that anti–MT1‐MMP treatment abrogates an increase in IL‐17 production in draining lymph nodes following anti‐TNF treatment. Whether this is indeed caused by reduced production of IL‐12/IL‐23p40 by macrophages needs further investigation, but the observation is clinically important since anti–MT1‐MMP treatment could potentially be used as an alternative to IL‐17 blockade to enhance the response to TNF inhibitors 42.

In conclusion, the findings presented herein suggest that MT1‐MMP constitutes an attractive target by which to halt the progression of joint damage in RA and to improve treatment outcomes in these patients, especially in those with an inadequate response to anti‐TNF therapy. Current developments in the treatments of RA include targeting cytokines or T or B lymphocyte subsets 43, 44, and the potential benefits of combining TNF inhibitors with other therapeutic agents should be carefully weighed against the increased risk of infection. MT1‐MMP blockade is promising, as it alters synovial cell behavior mediated through pathways different from those targeted by TNF inhibitors.

## AUTHOR CONTRIBUTIONS

All authors were involved in drafting the article or revising it critically for important intellectual content, and all authors approved the final version to be published. Dr. Itoh had full access to all of the data in the study and takes responsibility for the integrity of the data and the accuracy of the data analysis.

### Study conception and design

Kaneko, Williams, Itoh.

### Acquisition of data

Kaneko, Dransfield, Nixon, Sandison.

### Analysis and interpretation of data

Kaneko, Williams, Sandison, Itoh.

## ADDITIONAL DISCLOSURES

Authors Dransfield and Nixon are employees of Dyax Corporation.
